# Cathepsin K Inhibitors as Potential Drugs for the Treatment of Osteoarthritis

**DOI:** 10.3390/ijms26072896

**Published:** 2025-03-22

**Authors:** Leyre Brizuela, Rene Buchet, Carole Bougault, Saida Mebarek

**Affiliations:** Institut de Chimie et Biochimie Moléculaires et Supramoléculaires, Université de Lyon, Université Lyon 1, UMR CNRS 5246, 69 622 Villeurbanne Cedex, France

**Keywords:** cartilage, cathepsin K, chondrocyte, inflammation, joint, osteoarthritis, proteolytic enzymes

## Abstract

Links between cathepsin K and the pathophysiology of osteoarthritis (OA) can be established, not least because of the overabundance of cathepsin K in the serum of OA patients and the upregulation of cathepsin K in degraded cartilage in animal models of OA. Chondrocytes, chondroclasts, or osteoclasts contribute to the accumulated cathepsin K at the diseased osteochondral junction. After a general presentation of OA and cartilage physiology, as well as its degradation processes, we describe the function of cathepsin K and its effect on cartilage degradation via type II collagen cleavage. An overview of the most promising cathepsin K inhibitors is then presented, together with their in vitro effects. Although intensive research on cathepsin K inhibitors initially focused on bone resorption, there is growing interest in the potential of these drugs to prevent cartilage degradation. In this review, we summarize the pre-clinical and clinical trials that support the use of cathepsin K inhibitors in the treatment of OA. To date, no molecules of this type are commercially available, although a few have undergone clinical trials, but we believe that the development of cathepsin K inhibitors could broaden the therapeutic arsenal for the treatment of OA.

## 1. Introduction

**Figure 1:**
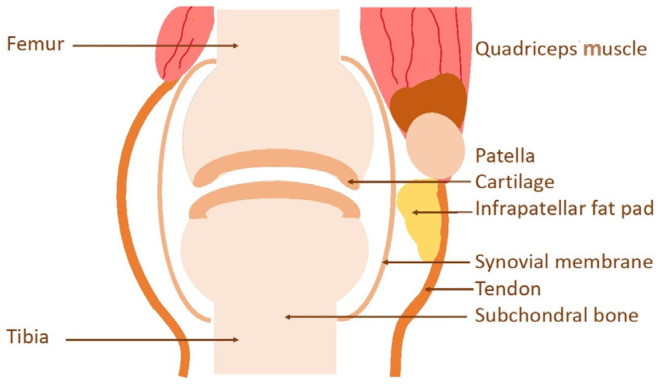
Knee joint scheme indicating, muscle, cartilage, synovial membrane, infrapatellar fat pad, tendon, and subchondral bone. Cartilage is not a vascularized tissue. Synovitis, tendon, muscle, infrapatellar fat pad, and subchondral bone are highly vascularized tissues, which can induce pain when they have lesions during OA.

Osteoarthritis (OA) is the most common joint disease [1,2,3,4,5], which leads to the degeneration and loss of articular cartilage, affecting over 500 million people, about 6%, worldwide [5]. It occurs in the knees, hips, and spine joints, as well in the hands and other non-weight-bearing articular sites [6,7,8,9,10,11]. OA results from the complex interactions of local and systemic factors, including aging, obesity, knee malalignment, loading of joints, and inflammation [9,11]. OA development is also affected by the female gender, especially during the decline of sex hormone levels in menopause, which may induce joint pains [10]. OA clinical symptoms are assessed by joint pain [12], radiographic assessment [7,13], and magnetic resonance imaging [7,14]. Cartilage lesions are not directly associated with pain since articular tissue is an avascular tissue [15]. The pain is induced from the lesions of synovitis, bone marrow, subchondral bone, osteophyte formation, abnormalities of infrapatellar fat pads, and ligaments, which have sensory nerves [16,17,18,19,20,21] (Figure 1).

Joint loading and repeated stress on cartilage can trigger joint inflammation [18]. Persistent inflammation produces intracellular or extracellular stimuli as damage-associated molecular patterns, which can contribute to the degradation of the extracellular matrix [17]. For instance, an infrapatellar fat pad can secrete adipokines, cytokines, growth factors, and lipid derivatives, which can contribute to the degradation of the extracellular matrix [18]. Synovitis inflammation is considered as a prognostic osteoarthritis marker and is proposed to explain the disconnection between radiography and patient symptoms [17].

The first link between cathepsin K and OA pathophysiology was established 25 years ago [22]. Cathepsin K was upregulated in the cartilage of knee joints in the first stages of the degradation process in a transgenic model of the OA (Del1 mice) process [23]. In this review, we focused on the inhibitors of cathepsin K as a possible drug therapy to repair OA cartilage. The methodology was based on a search in Pubmed and Pubchem by using keywords, including cathepsin K, chondrocyte, osteoarthritis, extracellular matrix, and cathepsin K inhibitors. A brief overview of the current treatments of osteoarthritis and promising approaches (Section 2), development of epiphyseal cartilage (Section 3) and articular cartilage (Section 4), degradation of cartilage (Section 5), function and level of expression of cathepsin K (Section 6), in vitro effects of cathepsin K inhibitors (Section 7), and pre-clinical and clinical trials on cathepsin K inhibitors (Section 8) are presented.

## 2. Current Treatments of Osteoarthritis and Promising Approaches

There is no drug treatment that will stop OA progression due to the complexity of its pathophysiology [24,25,26]. Most of the current OA treatments are associated with the relief of OA symptoms and pain (Table 1) [27].

Physical exercise (running [26], walking [27]), weight management [28], prescription of nonsteroidal anti-inflammatory drugs (celecoxib, diacerin, diclofenac, etoricoxib, ibuprofen, naproxen) [29,30,31,32,33,34], acetaminophen [33], duloxetine [29,33], and tramadol [33] can contribute to the relief of OA symptoms. Other treatments include the intra-articular injection of hyaluronic acid [35,36,37] or corticosteroids [26], as well as total joint replacement (Table 1) [38,39,40,41,42]. The accumulated clinical experience indicates that both joint function and pain shall be considered for an effective OA treatment [43]. This has stimulated an intensive research effort for several decades to evaluate alternate and promising strategies to treat OA (Table 2) [43,44].

**Table 1:** Current treatments of OA. NSAIDS: non-steroidal anti-inflammatory drugs.

Current Treatments	Types	Effects	Ref.
Physical exercise	Running	Relief of OA symptoms	[24]
	Walking	Relief of OA symptoms	[25]
	Weight management	Relief of OA symptoms	[26]
Acetominophen	Acetominophem, similar to NSAIDs	Analgesic and antipyretic	[31]
Duloxetine	Instead of NSAIDs and acetominophen	Release pain	[27,31]
NSAIDs	Celecoxib	Anti-inflammatory	[27]
	Diacerin	Anti-inflammatory	[27]
	Diclefenac	Antipyretic, anti-inflammatory	[28,29,30]
	Etoricoxib	Anti-inflammatory	[29]
	Ibuprofen	Anti-inflammatory	[32]
	Naproxen	Anti-inflammatory	[32]
Tramadol	Tramadol not used for first-time treatment	Opioid, relief of pain	[31]
Intra-articular injection	Hyaluronic acid intra-articular injection	Improves integrity of joints	[33,34,35]
	Corticosteroid intra-articular injection	Anti-inflammatory	[26]
Surgery	Joint replacement	Relieves pain and OA symptoms	[36,37,38,39,40,41]

Several encouraging findings on the inhibition of metalloproteases [43,45,46], intra-articular injections of chondroitin sulfate [46,47], oral administration of cordycepin [48], and strontium ranelate [49] reported better preservation of cartilage integrity. Another positive line of research focuses on catabolic and anabolic molecular pathways [50,51], such as cyclin-dependent kinase inhibitors [52]; inhibitors of CXCR4, a G-coupled receptor of chemokines [53,54]; melatonin [55]; and inhibitors of mitogen-activated protein kinase (p38MAPK) [56]. Several nutraceuticals may have potential benefits against OA [57,58,59,60,61,62,63,64,65,66,67], such as curcumin [59,60], flavonoids [61,62,63], ginseng [64], grapefruits [65], omega-3 fatty acids [66], and polyphenols [67], while the effects of vitamin D on OA are inconclusive [68,69,70]. Acupotomy improves subchondral bone, as indicated by an increase in the quality of bone morphometric parameters calculated from hematoxylin and eosin staining in knee OA rabbits [71,72]. In addition, it protects cartilage, as evidenced by a recovered Safranin-O staining in rabbits with knee OA [71,72]. To promote the intrinsic regenerative capacity of cartilage, the intra-articular injection of mesenchymal stem cells (MSCs) was the object of clinical trials and holds promise [73,74,75,76,77,78,79,80,81,82,83,84,85,86,87]. However, the number of MSCs [87] and the best source of MSCs [88] are still debated. The incorporation of a 3D cell-laden collagenous scaffold to replicate the native bone and cartilage extracellular matrix has the potential to repair bone and cartilage tissues [89,90]. The quality of cartilage repair with chondrocyte transplantation embedded in various types of scaffolds is affected by the nature of the scaffold, which may impact cartilage tissue regeneration [90,91]. Another line of research is the use of extracellular vesicles (EVs) [92]. EVs from MSCs are heterogeneous populations of particles with distinct characteristics [92]. EVs contain specific information from donor cells, have a strong ability to circulate in body fluid, and may contribute to the diagnosis of earlier stage OA [93,94,95,96,97,98]. The properties of EVs depend highly on the origin of the cells (bone MSCs, adipose tissue MSCs, synovial MSCs, and embryonic stem cells) from which they originate [93,98,99]. The injection of EVs as a cell-free therapy appears to be a promising approach to treat OA [100,101,102,103,104,105,106]. The injection of EVs may contribute to maintaining the chondrocyte phenotype [98,107,108] and have anti-inflammatory actions [107,108,109]. The intra-articular injection of EVs in animal models with OA reduced cartilage loss [94,110,111,112,113,114]. Scaffold materials, such as hydrogels, to fill in cartilage defects and retain MSC-EVs at the cartilage defect site can ensure precise cartilage repair and reverse OA progression [115,116].

**Table 2:** Promising treatments of OA. EVs—extracellular vesicles, MSCs—meschenchymal stem cells.

Promising Treatments	Types	Effects	Ref.
Acupotomy	Acupotomy	Improves subchondral bone in rabbit	[69,70]
Chondroitin sulfate	Chondroitin sulfate	Protective effects on joints	[44,45]
Chondrocyte transplantation	Chondrocyte transplantation	Cells did not reach cartilage	[88,89]
Cordycepin	Cordycepin	Chondroprotective effects on explants	[46]
Melatonin	Melatonin	Protects chondrocytes and cartilage	[53]
Inhibitors of chondrocyte receptors	Inhibition of Asporin	May prevent cartilage degradation	[51]
	Inhibition of CXCR4	Activates cartilage progenitor cells	[51]
	Inhibition of DDR2	Prevents mice cartilage degeneration	[52]
Inhibitors of metalloproteases	Inhibitors of metalloproteases	Proposed as a treatment of OA	[42,43]
Intra-articular injection of EVs	Intra-articular injections of EVs	Reduces cartilage loss in animals	[92,108,109,110,111,112]
Intra-articular injection of MSCs	Intra-articular injections of MSCs	Clinical trials hold promise	[71,72,73,74,75,76,77,78,79,80,81,82,83,84,85]
Nutraceuticals	Curcumin	Reduces pain but less than NSAIDs	[57,58]
	Flavonoids	More clinical trials are needed	[59,60,61]
	Ginseng	Anti-oxidant and anti-inflammatory	[62]
	Grapefruit	Chondroprotective effects on rat OA	[63]
	Omega-3 fatty acids	No changes in OA parameters	[57,64]
	Polyphenols	More clinical trials are needed	[65]
	Vitamin D	Inconclusive	[66,67,68]
Strontium ranelate	Strontium ranelate	Reduces the progression of dog OA	[47]

## 3. Development of Epiphyseal Cartilage

**Figure 2:**
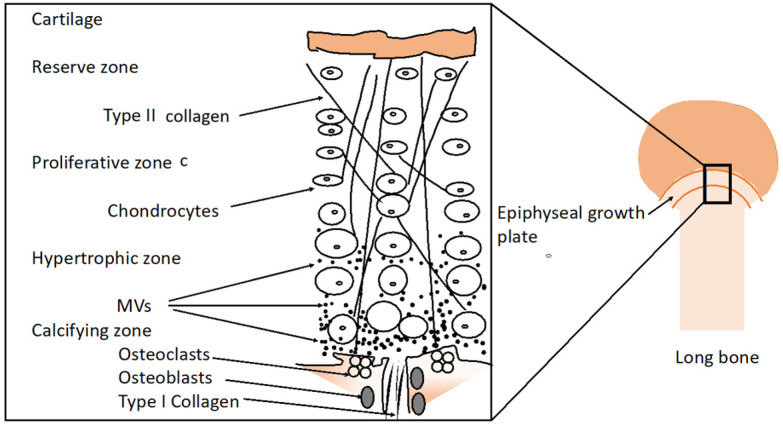
The epiphyseal growth plate is divided into the reserve zone, proliferative zone, hypertrophic zone, calcifying zone, and bone metaphysis. In the proliferative zone, which is enriched in type II collagen, cells are divided and form columns called longitudinal septa, which are separated by adjacent cell columns called transverse matrix septa. Matrix vesicles (MVs) (as indicated by black filled circles) are secreted when chondrocytes become hypertrophic in the hypertrophic zone. The perichondrium and the developing bone center are within the border of the calcifying zone enriched in osteoblasts, osteoclast, and type I collagen. Taken from [124].

A short overview of the development of epiphyseal cartilage is introduced to clarify the distinct functions of chondrocytes during endochondral ossification [117,118,119]. The chondrocytes are essential for producing extracellular matrix [120,121,122,123]. Chondrocytes produce and maintain the cartilage matrix [118]. In addition, hypertrophied chondrocytes calcify the cartilage in the growth plate cartilage during endochondral ossification [117,119]. The developing epiphyseal cartilage can be divided into three distinct zones: reserve or resting zone, proliferative zone, and hypertrophic zone (Figure 2) [2,117,118,119].

As chondrocytes become hypertrophic, their volume is increased five- to twelve-fold [2,123], and they cannot proliferate but they can mineralize the cartilaginous matrix [119]. The biogenesis of matrix vesicles (MVs) coincides with the sequence of events leading to apoptosis or programmed cell death of hypertrophied chondrocytes [119] (Figure 2). The discovery of vesicles at epiphyseal cartilage supports the theory that MVs initiate mineralization [119,125,126,127,128,129,130,131,132,133,134]. More specifically, MVs strongly bound to collagen form apatite in their lumina [135]. Both hypertrophied chondrocytes and MVs highly enriched in tissue non-specific alkaline phosphatase (TNAP) are able to hydrolyze pyrophosphate, an inhibitor of apatite, to sustain the mineralization process [119]. The cartilage becomes calcified and a newly vascularized bone tissue is formed. Hypertrophic chondrocyte differentiation is associated with high TNAP activity, and the synthesis and secretion of type X collagen follows that of type II collagen [2,136]. The expression of type I collagen by hypertrophic chondrocytes might be associated with differentiation into osteoblast-like cells [137,138,139,140]. The length of the hypertrophic zone is maintained by a balance between the rate at which chondrocytes enter the hypertrophic phase and the rate at which vascular endothelial cells invade cartilage at the chondro-osseous junction (Figure 2). The hypertrophic chondrocytes synthesize vascular endothelial growth factor (VEGF) to induce vascular invasion of the epiphyseal cartilage [141]. Besides this endochondral ossification, there is a so-called intramembranous ossification, where bones develop from mesenchymal cellular condensations, which subsequently directly undergo osteoblast differentiation [141] without the involvement of any chondrocytes.

## 4. Articular Cartilage

The articular cartilage has only one cell type: chondrocytes [116]. The chondrocytes, which abundantly secrete cartilaginous matrix [119], occupy about 1–2% of the total tissue volume (up to 5%) [2,120,121,122,123]. Cartilage, which is avascular, is essentially a matrix containing 60–80% water and 15–22% wet collagen fibers (mainly type II collagen, non-collagenous proteins, and glycosaminoglycans), and the remaining wet weight (4–7%) consists of hydrophilic proteoglycans and glycoproteins [120,142,143]. A particular pericellular matrix containing type VI collagen is also produced by embedded chondrocytes [143]. Healthy articular chondrocytes maintain the complete extracellular matrix by secreting type II collagen, proteoglycans, and related macromolecules. They respond to the physical properties of the cartilage extracellular matrix and the mechanical forces exerted on them during joint loading [2,144]. Glycosaminoglycan, aggrecan, and small proteoglycan core proteins, with shorter life spans than collagen, are replaced more often than collagen in response to mechanical loading [145,146,147]. Primary cilia located on the chondrocyte surface and mechanosensitive receptors serve as sensors for the chondrocytes to adapt their metabolic activity in response to mechanical loading [145]. Once bone is formed, the healthy chondrocytes in the articular cartilage do not undergo hypertrophied differentiation such that no calcification is induced under physiological conditions [148,149].

## 5. Degradation of Cartilage

### 5.1. Pathological Mechanisms of OA

OA affects the articular cartilage, subchondral bone, ligaments, capsule, synovial membrane, and muscles, which finally lead to joint failure [2,3,4]. The joint pain is not a uniform accompaniment to structural change caused by the cartilage but is related to the interactions with vascular tissues [15,16,17,18,19,20,21,150]. Pain in OA was reviewed [15,16,17,18,19,20,21,151,152] and will not be further reported in this review. The abnormal differentiation of chondrocytes toward hypertrophied chondrocytes or the osteoclast-like phenotype, as well the chondrocyte apoptosis or senescence [153], which is induced by excessive mechanical loading, may lead to changes in the composition and structure of the cartilage matrix [144,154,155,156,157]. During the late stage of OA, hypertrophic chondrocytes express genes associated with the calcification of cartilage, including type X collagen and runt-related transcription factor 2 (RUNX2) [158]. In this respect, the type X collagen is a diagnostic factor for knee OA in humans [159]. The inactivation of Wnt signaling pathway, which can be affected by sclerostin [160,161], Wnt-16 [160,162,163,164], and Dickkopf-1 (Dkk1) [165], may favor hypertrophy [166]. Among the integrins, which connect chondrocytes with the extracellular matrix, integrin α_5_β_1_ [167,168] integrates mechanical cues that induce inflammation [169] and catabolism [170]. Not only local cytokines (Interleuline-1β (IL-1β), RGD peptides, interleuline-4 (IL-4), sclerostin, and Dkk1) but also hormones (i.e., estrogen, heparin-binding EGF-like factor) regulate chondrocytes subjected to loads [147]. The chondrocyte apoptosis, induced by IL-1β, tumor necrosis factor, nuclear factor-kappa B, Wnt, microRNA, and oxidative-stress-signaling pathways can contribute to the degradation of the extracellular matrix [171]. Excessive loading cycles can trigger the abnormal differentiation of chondrocytes, which induce surface fibrillations and deep fissures associated with the exfoliation of cartilage fragments [171]. Taken together, excessive loading cycles may contribute to the expansion of calcified cartilage and the disturbance of articular cartilage homeostasis [172,173,174].

Concerning the mineral composition of calcified cartilage, a distinction shall be made between calcium pyrophosphate (CPP) crystals and apatite [175]. The CPP crystals occur in acute inflammatory arthritis (formerly known as pseudo gout) or in chronic CPP crystal arthritis [176,177], whereas OA instead deals with apatite. Apatite deposition in the cartilage during OA recapitulates a program of endochondral bone formation induced by hypertrophied chondrocytes [174], which may correspond to the advanced stage of OA [178]. Hypertrophied chondrocytes, senescent chondrocytes, and synovial fibroblasts contribute to the progression of OA [157]. It is still unclear whether hypertrophied chondrocytes or senescent chondrocytes come first to initiate OA [157]. An increased production of VEGFs, including VEGF121, VEGF165, and VEGF189, was observed in OA [179]. VEGFs secretion contributes to the chondrocyte differentiation, vascular invasion, and calcified cartilage expansion [179]. Angiogenesis triggers blood vessel growth, disrupts the osteochondral junction, and creates channels from subchondral bone spaces into noncalcified articular cartilage [180]. Blood vessels in joint OA can invade non-calcified cartilage [181], which facilitates the penetration of osteoclast precursors, and finally forms an ossification center [182,183]. Endothelial cells proliferate within the OA synovium [184,185,186]. Inflammation drives synovial angiogenesis through macrophage activation [187] and induces chondrocyte apoptosis [188]. Activated fibroblast-like synoviocytes in the OA synovium secrete cytokines, growth factors, metalloproteases (MMPs), and tissue inhibitors of metalloproteinases (TIMPs), which contribute to the macrophage activation and stimulate catabolic pathways in chondrocytes [188].

### 5.2. Enzymatic Degradation of Cartilage

The degradation of cartilage is initiated by the enzymatic activities of several proteases, including aggrecanases and collagenases, as ADAMs (disintegrin and metalloproteinases), ADAMTS (disintegrin and metalloproteinases with thrombospondin motifs), and MMPs [189]. The expressions of aggrecanases, such as ADAMTS-4 and ADAMTS-5 (previously named Aggrecanase 1 and Aggrecanase 2), are altered in OA [189,190,191]. They act on aggrecans, which are the major cartilage matrix components. In addition, ADAMTS-7 and ADAMTS-12, which contribute to cartilage matrix catabolism, and ADAM9, ADAM10, and ADAM12, which are involved in chondrocyte differentiation and proliferation, are overexpressed in OA [188,189]. Not all the enzymes overexpressed in OA may impair cartilage, for instance, ADAMTS-2, ADAMTS-3, and ADAMTS-14, may instead promote matrix anabolism [192]. Regarding the MMP family, elevated levels of MMP-3 and MMP-13, which act preferentially in the other major cartilage component type II collagen, are found in OA [189]. In OA, the excess of TIMPs do not increase to the same extent as those of MMPs, which may result in a cartilage breakdown [193]. It was proposed that inhibitors of collagenases, including MMP-1 (preferred substrate is type III collagen), MMP-2 (interstitial collagenase that degrades types I and IV collagen), MMP-8 (preferred substrates are types I and III collagen), MMP-9 (termed as gelatinase-B, which cleaves gelatins, elastins, aggrecans, and types IV and V collagens), and MMP-13, may have the therapeutic potential to prevent cartilage degradation (Table 2) [194].

Cathepsin K is one of the few extracellular proteolytic enzymes capable of degrading native fibrillar collagen [195]. Cathepsin K may contribute to the progressive destruction of articular cartilage in OA [195,196,197,198] and be a biomarker for monitoring the progression of joint destruction in OA [199]. The following chapters provide more insight into its function, level of expression, design of cathepsin K inhibitors, in vitro and in vivo findings, and preclinical trials.

## 6. Functions of Cathepsin K

### 6.1. Involvement of Cathepsin K in Osteoarthritis Physiopathology

The first link between cathepsin K and OA pathophysiology was established 25 years ago [22]. Cathepsin K is upregulated in the cartilage of knee joints in the first stages of the degradation process in a transgenic model of the OA (Del1 mice) process [23]. These mice possess six copies of a transgene with a small deletion mutation engineered into the mouse type II collagen gene [23]. Likewise, transgenic UTU17 mice with constitutive overexpression of cathepsin K [200] naturally developed synovitis and articular cartilage degradation [201]. Moreover, they developed early osteopenia in diaphyseal cortical bone and metaphyseal trabecular bone [202]. In contrast, cathepsin K-knockout mice exhibited delayed cartilage destruction in a model of destabilization-induced OA [203] and after anterior cruciate ligament transection (ACLT) [204] when compared with wild-type counterparts. Furthermore, a cathepsin K-knockout mice model with the destabilization of medial meniscus (DMM) also showed slower articular cartilage degradation and higher subchondral bone volume [205]. Cathepsin K is also involved in cartilage collagen degradation in naturally occurring equine OA [206] by degrading type II collagen [207].

### 6.2. Function and Level of Expression of Cathepsin K

During skeletal development, the strongest expression of cathepsin K is detected in the osteoclasts of bone following the onset of osteoclast differentiation [199,208]. First produced in an inactive zymogen form, its maturation occurs intracellularly in the osteoclasts on the bone surface, which shows intense enzyme activity [209]. During endochondral ossification in long bones, cathepsin K expression is localized at the osteochondral junction of growth cartilages in osteoclasts, but also in hypertrophic chondrocytes, and possibly in chondroclasts [199,210,211,212]. The upregulation of cathepsin K expression is associated with cartilage degeneration and disease progression in a mouse OA model [23]. Its expression is localized near sites of cartilaginous matrix degradation in both the proliferative and the calcified zone [23]. In agreement, the chondrocytes of OA patients overexpress cathepsin K at both the mRNA and the protein level in the superficial zone and damaged areas in relation to the severity of the OA [213,214]. In addition, high levels of cathepsin K mRNA are also detected within OA synovial tissue [22]. This overabundance of cathepsin K can even be detected in the circulatory system since its level is increased in the serum of OA patients [215]. In chondrocytes, Cathepsin K may also be involved in the gene and protein expressions of the chondrogenic markers type II collagen and aggrecan [216].

Cathepsin K is the key enzyme for osteoclastic activity: bone resorption [217,218,219,220]. It is one of the 11 cysteine cathepsins belonging to the family of papain-like cysteine peptidases [221,222,223,224]. Cathepsin K is the only one able to cleave the triple helix of collagen molecules at multiple locations [225]. During bone remodeling, its activity is crucial, even if it is supplemented by the collagenases of the MMP family [226,227,228]. Cathepsin K activity was reviewed in detail [222,224]. For bone matrix resorption, its main substrate is type I collagen [229,230], but in the context of OA, cathepsin K is a potent catalytic enzyme of cartilage degradation by the cleavage of type II collagen [207,231,232,233,234]. This proteolytic activity can be evaluated by the detection of the C-terminal neo-epitope generated. Such cartilage breakdown products are found in the lesion sites of OA cartilage [207,232,233], but also in the urine in a guinea pig OA model [235]. The stability and collagenolytic activity of cathepsin K is enhanced by complex formation with glycosaminoglycans [236,237,238]. These glycosaminoglycans, especially chondroitin [239,240,241,242,243] and keratan sulfates [244,245], are fragments of aggrecan aggregates, which are particularly present in the cartilage extracellular matrix. They are produced by self-activation through the cleavage of aggrecan by cathepsin K [246]. Another mechanism for amplifying the degradation process is that cathepsin K is also activated by the N-terminal telopeptides of type II collagen [231].

## 7. Design and In Vitro Effects of Cathepsin K Inhibitors

**Figure 3:**
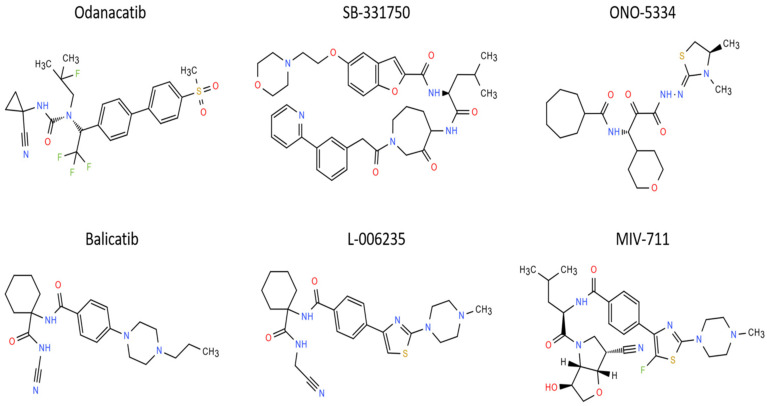
Chemical structures of the most promising cathepsin K inhibitors for treating OA.

For many years, the strong development of cathepsin K inhibitors has occurred with the aim of using them as therapeutic agents to treat osteoporosis [219,247,248,249,250,251,252,253]; OA [3,217,254,255]; and other diseases, including atherosclerosis, blood pressure disregulation, obesity, and cancer [223]. According to the ChEMBL database, there are around 280 compounds identified as cathepsin K inhibitors with a Ki ≤ 50 nM. One of the main problems with these compounds is their lack of selectivity against other cathepsins, such as cathepsins B, L, and S, which stimulated the development of selective inhibitors specific to cathepsin K [256,257]. There are several methods used to screen the inhibitors of cathepsin K, including the determination of enzymatic activity [258], cytochemical assays [259], ELISA detection of cleavage of type II collagen [260], and in vivo fluorescence reflectance imaging in animal models [261]. A short overview of the most promising cathepsin K inhibitors, with their potential applications to treat OA, is presented (Figure 3). Cathepsin K inhibitors are usually tested on chondrocytes to assess their eventual therapeutic effect to prevent cartilage degradation [262]. 6-shogaol, the most active ginger derivative, blocked TLR4-mediated innate immune responses and MMP induction in chondrocytes [262]. These findings suggest the potential for ginger derivatives’ use against cartilage and bone degradation [262]. Racemic cathepsin K inhibitors inhibited the cathepsin K activity in dedifferentiated chondrocytes [263]. A cathepsin K inhibitor obtained after a lead optimization of MK-1256 (Figure 3) reduced the levels of urinary C-telopeptide of collagen type I in a dog model of OA.

Derivatives of dipeptide- and peptide-containing groups, such as ketone, aldehyde, ketoamide, nitrile, and azanitrile, were developed as inhibitors of cathepsin K [264,265]. Among these electrophilic groups, the addition of a nitrile group to form a thioimidate adduct after a liaison with the catalytic residue Cys-25 of cathepsin K allows for reversible inhibition of the enzyme [263,266]. Another strategy was implemented using an alkyne molecule as a modified electrophile to induce an irreversible inhibition [267]. In addition, 1,3,4-oxadiazole derivatives have many applications in medicinal chemistry, including anti-inflammatory activity [268,269], analgesics [269], antimicrobial activity [270], antiparasitic activity [271], and anti-tumor activity [272,273,274]. For instance, raltegravir (antiretroviral) [274] and nesapidil (vasodilator) [275] are available on the market as drugs. To date, only one article has reported keto1,3,4-oxadiazole derivatives as cathepsin K inhibitors [276]. Heterocyclic building blocks allowed for designing effective cathepsin K inhibitors [277,278,279].

A series of piperidamide-3-carboxamide derivatives were synthesized as inhibitors of cathepsin K with an IC_50_ value of 0.08 μM [280]. Molecular docking analyses showed that one of the best compounds, H-9, reacts thanks to several hydrogen bonds and hydrophobic interactions with the main active residues of the active site of cathepsin K [280]. Using osteoclast RAW264.7 cells, the H-9 compound has anti-resorption effects on bone slices similar to those of MIV-711 [280].

Proline-based peptidomimetic inhibitors selectively blocking cathepsin K were developed [281]. The most active had a high affinity (K_i_ = 7.3–50.1 nM) for cathepsin K and did not inhibit other cathepsins [281]. This specific inhibitor has two substituents: trifluoromethylpyrazole and 4-methylproline in positions P3 and P2 [281]. Basic lysosomotropic inhibitors of cathepsin K can accumulate in the cells with a high lysosome content due to their basic and lyophilic properties [282]. Lysosomotropic cathepsin K inhibitors were able to inhibit the cathepsin K activity in synovial fibroblasts and stimulate periosteal bone formation in monkeys, suggesting that these molecules can prevent cartilage degradation [283]. Odanacatib (ODN, MK-0822) (Figure 3) acts as a reversible inhibitor: its action is on the cysteine part of the active site [284]. In vitro and in cellulo ODN is highly selective for cathepsin K, with values ~300-fold higher than cathepsin S and >1000-fold higher than other human cathepsins, including cathepsins B and L [284]. Due to its potency and selectivity, ODN is highly effective in humans [285]. Several structurally different cathepsin K inhibitors were developed, such as SB331750 (Figure 3) [286] and ONO-5334 (Figure 3) [287]. These inhibitors are also effective at increasing the bone mass in the ovariectomized rat model (OVX) at relatively high doses.

## 8. Pre-Clinical and Clinical Trials on Cathepsin K Inhibitors

The cathepsin K inhibitors could be a therapeutic option for the treatment of several bone and joint diseases where an osteoclast dysfunction occurs, especially osteoporosis [288]. There is also the possibility to use cathepsin K inhibitors to treat OA or cancer-derived osteolytic bone metastasis induced by breast cancer or colon cancer [219,289]. Currently, there are no FDA-approved cathepsin K inhibitors, but several molecules have been tested in pre-clinical studies and/or clinical trials [290,291,292]. Here is a summary of the most studied molecules through the last 15 years that reached clinical trials (Table 3 and Figure 3).

One of the oldest tested inhibitors is ODN (MK-0822), which was initially designed to treat osteoporosis and significantly reduced bone fracture rates in a 5-year trial (Table 3) [293]. However, it was associated with an increased risk of cardiovascular events, specifically stroke in postmenopausal women with osteoporosis [294]. Similar clinical phase III findings were also observed for men [295]. Further development on ODN to treat osteoporosis was thus stopped. One of the most studied cathepsin K inhibitors for the treatment of OA is balicatib (AAE581) (Table 3). Balicatib was tested in patients that suffered from knee OA. Even if an improvement of bone and cartilage structure was observed, the study was suspended due to important side effects in patients, such as skin rashes or dermal fibrosis (NCT00170911, NCT00100607, and NCT00371670) (Table 3) [296]. Moreover, skin-related side effects were also observed in a multicenter clinical trial in North America and Europe that evaluated balicatib for the treatment of osteoporosis. A total of 9 patients out of 709 (1.3%) experienced morphea-like symptoms (skin hardening and dermal fibrosis). These symptoms were dose-related and completely disappeared in eight patients and partially in one patient after treatment discontinuation [297]. Of note, balicatib was able to induce bone formation (spine and femur) in ovariectomized monkeys, an experimental model of osteoporosis [298].

Two other cathepsin K inhibitors were efficient at treating OA symptoms in non-murine in vivo models. SB-553484, a strong cathepsin K inhibitor (Ki = 0.14 nM for human-cathepsin K), was able to significantly reduce cartilage degradation in female beagle dogs after a partial medial meniscectomy (Table 4) [299].

The cathepsin K inhibitor AZ12606133 efficiently decreased joint pain and the urine levels of CTXII (cross-linked C-telopeptides of type II) in a spontaneous model of OA in guinea pigs (Table 4) [235]. Other approaches, such as the intra-articular delivery of disease-modifying OA drugs, are expected to be among the most successful in the treatment of early post-traumatic OA. For example, it was recently demonstrated that soft materials, such as hydrogel, are able to encapsulate a cathepsin K inhibitor, L-006235 [300], and showed sustained drug release in the knee joint of DMM mice. Notably, L-006235 hydrogel significantly reduced the cartilage degeneration in both control and running mice (Table 4) [301]. In agreement with these results, the oral administration of L-006235 to ACLT-mice and rabbits protected them from cartilage damage (Table 4) [204].

Recently, a strong and selective inhibitor of cathepsin K, MIV-711, attenuated joint degradation in rabbits and dogs subjected to ACLT (Table 4) [302]. MIV-711 was considered as a good candidate to treat OA because of its oral availability, safety, and high tolerance by healthy subjects (Table 4) [303]. A first phase IIa clinical trial significantly reduced the medial femoral cartilage thickness after 26 weeks of treatment, while NRS pain scores with MIV-711 were not statistically significant with respect to the placebo (Table 3) [304]. This was challenged by another trial investigation with 119 patients and by looking predominantly at unilateral knee pain [305] in contrast with the inclusion of pain from non-joints [304]. There was a significant reduction in OA unilateral knee pain due to the MIV-711 treatment, with beneficial effects on the cartilage (Table 3) [305]. Recently, MIV-711 received a rare pediatric disease designation and orphan drug designation by the FDA for the treatment of the rare childhood Legg–Calvé–Perthes disease [301,302,306].

**Table 3:** Clinical trials on cathepsin K inhibitors. INN: international nonproprietary name, NRS: numeric rating scale for pain, OA: osteoarthritis.

Inhibitors	INN	Target Diseases	Clinical Trial	Benefits	Side Effects	Ref.
Odanacatib	MK-0822	Osteoporosis	NCT00620113 NCT00729183 NCT00529373 NCT01120600	Reduction in bone fracture rates	Increased risk of cardiovascular events	[293,294,295]
Balicatib	AAE581	Knee OA or osteoporosis	NCT00371670 NCT00170911 NCT00100607	Improvement of bone and cartilage structure	Skin rashes and dermal fibrosis	[296,297]
MIV-711		Knee OA	NCT02705625	Reduction in medial femoral cartilage thickness	No change in NRS pain scores compared with placebo	[303,304]
MIV-711		Knee OA	NCT03037489	Reduced change in bone area Greater reduction in unilateral knee joint pain	No difference in cartilage thickness	[305]

**Table 4:** In vivo findings on cathepsin K inhibitors. ACLT: anterior cruciate ligament transection, CTX-I: C-telopeptide collagen type I, CTX-II: C-telopeptide collagen type II, DMM: destabilization of medial meniscus, INN: international nonproprietary name, OA: osteoarthritis.

Inhibitors/ INN	Target Diseases	Admnist.	In Vivo Models	Benefits	Side Effects	Ref.
Balicatib/AAE581	Osteoporosis	Gavage	Ovariectomized monkeys	Stimulation of periosteal bone formation (spine and femur)	Not described	[298]
SB-553484	Knee OA	Oral	Female beagle dogs with partial medial meniscectomy	Reduction of cartilage degradation; reduced urine levels of CTX-I and CTX-II	Not described	[299]
AZ12606133	OA	Oral	Guinea pig (spontaneous model)	Decreased joint pain; reduced urine levels of CTX-II	Not described	[235]
L-006235	Knee OA	Encapsulated in hydrogel and knee insertion	DMM mice	Reduced cartilage degeneration	Not described	[300]
L-006235	Knee OA	Oral	ACLT mice and rabbits	Protection of cartilage damage	Not described	[301]
MIV-711	Knee OA	Oral	ACLT rabbits and dogs	Attenuation of joint degradation	Well tolerated	[302]

## 9. Concluding Remarks

The facts that chondrocytes express cathepsin K [213,214], that the cartilage surface pH is more acidic (around 6.2–5.5) in the fissured cartilage surface than that on the normal cartilage surface (around 7.1) [196], and that cathepsin K can degrade type II collagen [207,231,232,233,234] indicate that a cathepsin K inhibitor may be a suitable drug therapy to prevent cartilage degradation during OA. It is still unclear whether the secreted cathepsin K found in the OA joint cartilage originate mostly from chondrocytes; from chondroclasts; or from osteoclasts, which can penetrate into the damaged cartilage [306]. To date, there are no cathepsin K inhibitors that have entered the drug market, despite the fact that a few molecules reached up to phase III and other clinical trials [291]. The design of cathepsin K inhibitors could serve to treat OA. In addition, the possibility that cathepsin K inhibition could have an analgesic effect shall be further explored to extend the drug arsenal to treat OA pain [307].

## Data Availability

Not applicable.
